# Big Data, Data Science, and Causal Inference: A Primer for Clinicians

**DOI:** 10.3389/fmed.2021.678047

**Published:** 2021-07-06

**Authors:** Yoshihiko Raita, Carlos A. Camargo, Liming Liang, Kohei Hasegawa

**Affiliations:** ^1^Department of Emergency Medicine, Harvard Medical School, Massachusetts General Hospital, Boston, MA, United States; ^2^Division of Rheumatology, Allergy, and Immunology, Department of Medicine, Harvard Medical School, Massachusetts General Hospital, Boston, MA, United States; ^3^Department of Epidemiology, Harvard T.H. Chan School of Public Health, Boston, MA, United States; ^4^Department of Biostatistics, Harvard T.H. Chan School of Public Health, Boston, MA, United States

**Keywords:** big data, data science, causal inference, the ladder of causation, machine learning

## Abstract

Clinicians handle a growing amount of clinical, biometric, and biomarker data. In this “big data” era, there is an emerging faith that the answer to all clinical and scientific questions reside in “big data” and that data will transform medicine into precision medicine. However, data by themselves are useless. It is the algorithms encoding causal reasoning and domain (e.g., clinical and biological) knowledge that prove transformative. The recent introduction of (health) data science presents an opportunity to re-think this data-centric view. For example, while precision medicine seeks to provide the right prevention and treatment strategy to the right patients at the right time, its realization cannot be achieved by algorithms that operate exclusively in data-driven prediction modes, as do most machine learning algorithms. Better understanding of data science and its tasks is vital to interpret findings and translate new discoveries into clinical practice. In this review, we first discuss the principles and major tasks of data science by organizing it into three defining tasks: (1) association and prediction, (2) intervention, and (3) counterfactual causal inference. Second, we review commonly-used data science tools with examples in the medical literature. Lastly, we outline current challenges and future directions in the fields of medicine, elaborating on how data science can enhance clinical effectiveness and inform medical practice. As machine learning algorithms become ubiquitous tools to handle quantitatively “big data,” their integration with causal reasoning and domain knowledge is instrumental to qualitatively transform medicine, which will, in turn, improve health outcomes of patients.

## Introduction

### Can “Big Data” Transform Medicine?

By now, it is increasingly recognized that “big data will transform medicine into precision medicine.” However, data by themselves are useless (1). Data alone are insufficient to achieve precision medicine, let alone to address its defining *cause-and-effect* questions—i.e., identifying the optimal prevention or treatment strategy, the subgroup of patients who would benefit, and when they would benefit most (2). To become useful, data should be queried, analyzed, and acted upon. It is causal reasoning, knowledge, and algorithms—not data—that prove transformative.

### Modern Statistics and Causal Inference in the Past Century

In the recent history of science, statistics have occupied a privileged position in learning from data and epistemically justifying inductive reasoning (3). However, in the 1920s, the founders of modern statistical science—such as Ronald A. Fisher—declared that statistics could study causes and effects (i.e., causal inference) by using data from randomized experiments, but not from observational studies (4). Nevertheless, clinicians and researchers continued to leverage observational data in order to tackle complex causal questions—e.g., the effect of prenatal factors on bronchiolitis (5), lifestyle factors on asthma (6), and environmental exposures on lung function (7)—particularly when randomized experiments were unethical or otherwise infeasible. Despite these efforts, until recently, mainstream statistics has provided clinicians and researchers with few approaches to explicitly articulate, let alone to answer, causal questions (1, 8). Consequently, every student has learnt that “correlation is not causation” (with good intention) and causal vocabulary in observational research has been virtually prohibited in some major journals (9, 10). These have classified an entire category of questions (i.e., cause-and-effect questions) in the medical science as not amenable to formal quantitative inference.

### Data Science in the Twenty-First Century

In the current “big data” era, there exists a rapidly-increasing volume, variety, and velocity of health information [e.g., clinical, electronic health record, and biometric (from wearable devices) data]. In parallel, the recent emergence of “data scientists”—most of whom are not formally-trained in traditional statistical science—has brought a neutral mindset that does not *a priori* preclude them from answering causal questions in observational studies (11). These scientists coined a term, “data science” or “health data science” as a component of medicine (see Glossary in Table 1), to refer to their realm, which is widely embraced by both of the industry and academia (11). The availability of “big data” and the influx of data scientists—alongside of the advent in epidemiological and statistical methods—present opportunities to unleash the wealth of “big data” to address the fundamental causal questions in precision medicine.

**Table 1:** Glossary.

Causal effect	In this article, causal effects refer to average causal (or treatment) effects rather than individual causal effects. In a binary exposure situation (e.g., treatment yes vs. no), it is the average difference between two counterfactual outcomes under two different treatments across all individuals in the population. The effect can be represented with different measures—e.g., risk difference, risk ratio, and odds ratio
Causal graphs	A graphical tool for qualitatively encoding domain knowledge and *a priori* assumptions on the causal structure of interest. It consists of nodes [which represent random variables (e.g., exposure, outcome, confounders, mediators, colliders)] and edges (which represent their causal interrelations). It also qualitatively represents dependencies and independencies between the variables in the data. It is often referred as a causal directed acyclic graph (DAG)—“directed” because the edges imply a direction and “acyclic” because there are no cycles between nodes
Causal inference	The process of using data in a sample to infer cause-and-effect relationships in the target population of interest
Collider	A variable that is causally influenced by two or more variables. In a causal diagram, it is a node on which multiple directed edges “collide” (Figure 1A). Adjustment for a collider results in a non-causal association between exposure and outcome, leading to selection bias (e.g., birth-weight paradox)
Confounding	The structural definition of confounding is the bias secondary to common causes of exposure and outcome (i.e., the bias due to confounders). For example (Figure 1C), baseline severity is a common cause of the exposure and the outcome, which leads to confounding
Consistency	One of three identifiability conditions. Consistency means that the observed outcome for every exposed individual equals his or her (counterfactual) outcome if he or she had received the exposure. This condition requires a well-defined exposure or treatment
Counterfactual causal inference	Causal inference based on the framework of counterfactuals to identify and estimate causal effects. For a binary exposure situation (e.g., treatment yes vs. no), this framework presupposes the existence of two outcome states (i.e., two counterfactual outcomes) to which all individuals of the population could be exposed. Counterfactual framework encompasses several models, such as the Neyman-Rubin potential outcome model and Pearl's structural causal model
Data	Information that are collected through observation [e.g., through observational studies, randomized controlled trials, biobanks, biometrics, electronic health records (Figure 3)]
Data science	An interdisciplinary concept that extracts knowledge and insights from data, using theories and techniques from many fields including computer science, statistics, epidemiology, and other domain knowledge sciences (e.g., medicine). Its major tasks include association and prediction, intervention, and counterfactual causal inference (and description). In this article, data science and health data science are used interchangeably
Domain (or subject-matter knowledge)	The knowledge of specialists or experts in a particular field. In our situation, it represents clinical and biological knowledge (e.g., medicine, pediatrics, pulmonology)
Effect modification	The situation where the magnitude (i.e., quantitative) or the direction (i.e., qualitative) of the effect of exposure on the outcome differs depending on a third variable—the “effect modifier.” Effect modification is sometimes called an “interaction” in statistical science
Exchangeability	One of three identifiability conditions—the exposed and unexposed individuals are exchangeable with regard to their risk factors for the outcome. In a randomized controlled trial, randomization ensures that these risk factors are equally distributed. In an observational study (conditional) exchangeability can be achieved by adjusting for a sufficient set of confounders (i.e., no unmeasured confounding)
Identifiability conditions	Three conditions (consistency, exchangeability, and positivity) required to identify the average causal effect of interest from data. When three identifiability conditions hold true, an observational study can be conceptualized as a conditionally randomized experiment
Instrumental variable (IV) methods	An analytic approach that examines the causal effect of exposure on outcome. This approach replaces the exchangeability assumption (i.e., no unmeasured confounding) with an alternative set of IV conditions—the relevance, independence, exclusion criterion conditions, and monotonicity (Table 3). Commonly-used IVs in health data science are genetic variants (i.e., Mendelian randomization), provider preference, and access to treatment
Machine learning	Machine learning (particularly, statistical learning) refers a set of algorithms for modeling and understanding complex data. It encompasses many algorithms, such as supervised learning (e.g., lasso regression, random forest, boosting, neural network [or deep learning]) and unsupervised learning (e.g., clustering, principal component analysis). Some examples are summarized in Table 3
Mediation analysis	Causal mediation analysis is an approach that aims to tease apart the total effect, natural indirect (or mediation) effect, and natural direct effect by using a counterfactual framework. The natural indirect effect represents how much the outcome risk would change if patient were set to be exposed, but the mediator value were changed from the value it would take if unexposed to the level it would take if exposed. The natural direct effect represents how much the outcome risk would change if patient were set to be exposed vs. to be unexposed but for each patient the mediator value were kept at the level it would have taken in the absence of exposure
Mendelian randomization	An analytic approach that examines the causal effect of a modifiable exposure (e.g., physical traits, molecular biomarkers) on the outcome of interest by using genetic variants as IVs
Positivity	One of three identifiability conditions—the probability of receiving every value of treatment/exposure conditional on a set of covariates is >0 (i.e., positive). For example, if all individuals received the same treatment/exposure level (i.e., a violation of positivity), it would be impossible to estimate the average causal effect

### Goals of the Primer

In this primer, we (1) discuss the principles of data science and its major tasks based on the “ladder of causation” classification, (2) introduce the commonly-used data science tools, with a focus on causal inference, and (3) outline current challenges and future directions in the field of medicine. We also elaborate on how data science can pave the way toward the development of precision medicine, with common medical conditions as examples.

## Goals of Data Science and the Ladder of Causation

It is key to understand what data science is (and is not). Although data science is often characterized by its tools (e.g., machine learning), scientific disciplines are primarily defined by their questions and tasks. For example, we define astrophysics as the discipline that studies the behavior and physical properties of the universe, not as the discipline that uses telescopes. Accordingly, we organize questions and tasks of data science into three defining classes, according to the “ladder of causation” proposed by a computer scientist, Judea Pear: (1, 8) (1) association and prediction, (2) intervention, and (3) counterfactual causal inference. Table 2 summarizes the 3-level classification, together with corresponding scientific questions, assumptions, and tools. A similar classification scheme has also been developed in the field of epidemiology (11).

**Table 2:** Scientific questions, required information, and analytical methods of data science according to the ladder of causation.

	**Examples of scientific question**	Required information^*^	**Examples of analytics and tools**
Rung 1 association and prediction	- What are the risk factors for developing asthma? - What is the probability of developing asthma in a patient with a set of predictors?	- Risk factors/predictors - Outcomes	- Regression - Supervised machine learning algorithms (e.g., random forests, neural network/deep learning)
Rung 2 intervention	Will a new biologic agent decrease the rate of asthma exacerbation by 30%, compared to placebo?	- Eligibility criteria - Exposures/treatments - Outcomes	- Elementary statistics in RCTs (e.g., risk differences of the outcome) - Intention-to-treat analysis - Per-protocol analysis - Causal Bayesian network
Rung 3 counterfactual causal inference	What would be the preventive effect of a new drug had it been given to a group of patients with a set of characteristics?	- Eligibility criteria - Exposures/treatments - Outcomes - Observation period and temporality^†^ - Domain knowledge on the causal structure (e.g., confounders, mediators, colliders)	- Regression - Propensity score matching - Standardization/G-formula - IPW/MSM - Targeted learning - IV-methods/Mendelian randomization

*IPW/MSM, inverse probability weighing for marginal structure model; IV, instrumental variable; RCT, randomized controlled trial.*

* *For all tasks, no information bias (no measurement error or misclassification) and no model misspecification are required.*

† *The effect of interest must occur after the cause (and an expected delay) during an observation period*.

### Association and Prediction

The first task of data science is data-driven—association and prediction, which constitutes the first rung of the causal ladder. Association invokes exclusively probabilistic relationships between the variables within observed data. For example, in a cohort study, we say that recurrent wheezing in early childhood is associated with the development of asthma, when the probability of observing one variable depends on that of the other (or vice versa).

Prediction maps the derived probabilistic association to future data in order to forecast the conditional probability of outcome. It encompasses both relatively simple tasks [e.g., developing clinical risk scores, such as the Asthma Predictive Index (12)] and more complex ones [e.g., a polygenic risk score using millions of genetic markers to predict which patients are at higher risk of asthma (13)]. Analytical tools range from basic computations (e.g., correlation coefficients in multivariable regression models), to Bayesian networks, to supervised machine learning algorithms [e.g., random forests, neural network (or deep learning)] (Tables 2, 3).

**Table 3:** Major analytical tools used in data science.

**Analytics and tools**	**When to use?**	**What to look for?^**a**^**	**Advantages**	**Disadvantages**
Causal mediation analysis (14)	Counterfactual causal inference	- The models well-represent the hypothesized cause-and effect process that generates the data (e.g., temporal sequence) - A set of exposure-outcome, exposure-mediator, and mediator-outcome confounders (specified in a causal diagram) is adjusted in the models	- Identification of causal mechanisms (e.g., direct and indirect effects) - Interaction between the exposure and mediator accounted	- Interpretation of natural direct and indirect effects is complicated (Table 1) - Cross-world counterfactuals
Inverse probability weighting and marginal structural model (15)	Counterfactual causal inference	- Model specifications - Violation and quasi-violation of positivity assumption (e.g., small proportion of patients has a disproportionately high influence)	- Time-varying effects can be estimated - Modeling the exposure is often less complicated than modeling the outcome - Both conditional and marginal effects can be estimated - Inverse probability of censoring weighting can account for potential selection bias	- Methodologically complex - Sensitive to quasi-violations of positivity assumption
**Machine learning algorithms** **(****16****)**				
Lasso regularization	Association/prediction	- Identification of hyperparameter - Performance in a separate population (i.e., transportability)	- Automated covariate selection - Simple interpretability	Only linear relation can be accommodated
Neural network/deep learning	Association/prediction	- Sample size - Approaches for data pre-processing (e.g., normalization) - Approaches that address overfitting (e.g., dropout) - Transportability	- Large number of predictors and non-linear relations can be accommodated - Superior prediction performance in many complex tasks (e.g., imaging diagnostics)	- Large sample size is often needed - Explainability is limited (“black-box”) - Transportability to other domains is often limited
Random forest	Association/prediction	Same as neural network	- Applications to identification of heterogeneous treatment effects (causal forest)	- Transportability to other domains is often limited
Unsupervised learning (e.g., hierarchical clustering, k-means)	Description of data (e.g., dimensional reduction, clustering)	- Appropriateness of the chosen distance measure for the dataset - Consistency across the different hyperparameters (e.g., distance, number of clusters)	- Hypothesis-free - High-dimensional data can be mapped to a lower-dimensional space (i.e., greater interpretability)	- Hypothesis-generating in nature - Susceptible to hyper-parameters (e.g., distance, number of clusters chosen)
Mendelian randomization (or IV analysis) (17)	Counterfactual causal inference	Four IV conditions: (1) Relevance: strong correlation between genetic instruments and exposure(2) Independence: no association between instruments and exposure-outcome confounders(3) Exclusion restriction: instruments affect the outcome only through the exposure(4) Monotonicity assumption: increasing the number of effect alleles for an individual can only increase the level of exposure, and can never decrease it	- No-unmeasured- confounding assumption is not required - Only summary statistics of genome-wide association studies (i.e., no individual-level data) may be used	- Identification of appropriate instruments is often difficult - Estimated effect is limited to “compliers” - Only life-long effects are estimated - Variants-exposure association may be time-varying
Propensity score matching (18)	Counterfactual causal inference	- Model specifications - Covariate balance in the matched sample - Target population that is inferred from the matched sample	Simple interpretability	- Matched sample is often poorly-characterized - Time-varying effects cannot be estimated
**Randomized controlled trial**				
Intention-to-treat (ITT) analysis	Intervention	- Adherence to assigned treatment - Target population of interest - Differential loss to follow-up	- Interpretation is simple - Estimates the effect of treatment assignment, regardless of treatment actually received - May provide a more conservative causal estimate	- The causal estimate is not often the effect of interest in clinicians (i.e., ITT is agnostic about treatment decisions after the random assignment) - Target population may be ill-characterized
Per-protocol analysis	Intervention	- Adherence to assigned treatment - Post-randomization confounding (e.g., confounder-treatment feedback) - Target population of interest - Differential loss to follow-up	Estimates the effect of receiving the treatment as specified in the study protocol (if accounted for time-varying prognostic factors associated with adherence).	- Post-randomization time-varying factors are often unmeasured or unaccounted. - Target population may be poorly-characterized
Regression (19)	- Association/prediction - Intervention - Counterfactual - causal inference	- Model specifications - Consideration of effect modification	- Simple interpretability - Wide-spread use	- Only conditional effects (within the levels of covariates) can be estimated (i.e., not marginal effects) - Time-varying effects cannot be validly estimated
Standardization/g-formula (15)	Counterfactual causal inference	Model specifications	- Marginal effects can be estimated - Time-varying effects can be estimated (g-formula)	- Methodologically complex - Computationally heavy
Targeted learning using TMLE (20, 21)	Counterfactual causal inference	Standard identifiability conditions (Table 1)	- Use of machine learning that places minimal assumptions on the distribution of data and accommodate complex non-linear relationships - Semiparametric estimation that allows known asymptotic properties of bias and variance	- Methodologically complex

*IV, instrumental variable; TMLE, targeted maximum likelihood estimation.*

a *or any causal inference methods (except for IV-methods), the standard identifiability conditions (Table 1) are required*.

Machine learning algorithms excel in the association and prediction tasks. For example, this is what Alpha Go (a computer program that plays the board game Go) does when its deep learning algorithms learn the existent and simulated data of millions of Go games to determine which move is associated with the highest probability of winning (22). However, these algorithms have ongoing challenges, such as explainability (or “black-box” algorithms) (23), transportability (to different questions, populations, and settings), and particularly the lack of causal reasoning. Accordingly, association and prediction, along with the tools employed, are placed at the first rung of the progressively more sophisticated rungs of the ladder (1, 8).

### Intervention

The second task of data science is intervention. It constitutes the second rung of the causal ladder because it involves not only observing the data but also changing what we observe, according to our causal belief (or causal hypothesis). For example, suppose we are interested in a causal hypothesis that treatment with a biologic agent would decrease the frequency of severe asthma exacerbation. A very direct way to estimate the effect of treatment is to perform an experiment under carefully-controlled conditions, such as randomized controlled trials (RCTs). Under a set of major assumptions specific to interventions—e.g., perfect adherence to assigned intervention, no selection bias due to a differential loss to follow-up, and no post-randomization confounding [i.e., sequential exchangeability (24) (Table 1)], an RCT would yield a consistent estimate for the causal effect of interest. Besides, the stable unit treatment values assumption (SUTVA)—(1) no interference and (2) no multiple versions of treatment—is also vital for consistently estimating the causal effect of interest. For example, in a simple RCT to investigate a vaccine efficacy, SUTVA would be violated due to herd immunity (a spillover effect). Tools used for intervention tasks range from basic computations (e.g., risk differences by an intention-to-treat analysis) to more-complex analytical methods [e.g., causal Bayesian networks (25)] (Table 2).

Ideal RCTs that meet the assumptions above have been considered the “gold standard” for establishing causal inference (26). Why not conclude this review article here? Unlike A/B tests performed by information technology companies, RCTs in clinical research are often impossible to conduct for a number of logistical, practical, and ethical reasons (e.g., examining the causal effect of prenatal smoking exposures on health outcomes of the offspring). Most importantly, in precision medicine, we seek to make inferences from the existent data of a set of patients who are similar—in as many characteristics as possible—to the patients of interest. However, any interventional experiment cannot tackle “what if?” or retrospective questions (e.g., “what if this patient had received treatment *X* at time *t*?”) using the existent data that cohorts and consortiums possess. No experiment can remove medications from already treated individuals and measure their outcomes. For that reason, we must deploy a new set of tools to tackle these important questions.

### Counterfactual Causal Inference

The third task of data science—the final rung of the ladder—is counterfactual causal inference (Table 1). In the long history of human efforts to understand the meaning of “causality,” stretching back to the time of Aristotle (27), the origin of counterfactuals—a mode of causal reasoning—goes back to the philosopher David Hume in the 1700s. Hume defined causality to be: “if the first object had not been, the second never had existed” in his *An Enquiry Concerning Human Understanding* (28). By the beginning of the twenty-first century, a unified framework of quantitative causal inference (i.e., counterfactual outcome framework) was developed (15, 25, 29).

Counterfactuals are how humans naturally reason causal effects. We instinctively apply a possible-world semantics, and compare two outcomes: (1) the outcome—say, anaphylaxis (yes/no)—that would have been observed with a hypothetical treatment/exposure—say, new drug (yes/no)—vs. (2) the outcome that would have been observed without one. These two outcomes are referred to as counterfactual (or potential) outcomes because they represent world(s) that may not exist—i.e., counter-to-the-fact worlds (15). Then, the counterfactual definition of *individual* causal effect is the following: the treatment/exposure has a causal effect on the outcome if these counterfactual outcomes differ for the individual. Note that only one of these outcomes is observed for each individual (the outcome that corresponds to the treatment/exposure actually occurred in the individual), while the other outcomes cannot be observed. Because of the missingness, individual causal effects—as a general rule—cannot be identified. Instead, an aggregated causal effect—the *average* causal effect in a population—is used (Table 1) (15). Its definition is the following: a contrast of the proportions of outcome (e.g., anaphylaxis) that would have been observed (1) if all individuals had been treated/exposed (e.g., new drug) vs. (2) if all individuals had *not* been treated/exposed in the population of interest.

The counterfactual causal inference framework enables us to formulate causal questions, encode them in algorithms, and to identify average causal effects from data—even data from observational studies—under the identifiability conditions (Table 1). Its tools range from a relatively-simple ones (e.g., multivariable regression models adjusting for confounders) to more-advanced methodologies [e.g., inverse-probability weighting for time-varying treatments (15), targeted learning leveraging machine learning algorithms (20, 21); Tables 2, 3].

The primary difference between the first task (association and prediction) and third task (counterfactual causal inference) of data science is the role of domain knowledge, which in our situation is clinical and biological knowledge. Note that the former task invokes only the probabilities between the variables within data, the latter task cannot be completely defined by the probabilities in the factual world. Causal inference calls for domain knowledge not only to define counterfactual causal effects but to specify the causal structure of interest—e.g., the relationship between the treatment, outcome, confounders, mediators, and colliders (15) (Table 1).

**Figure 1:**
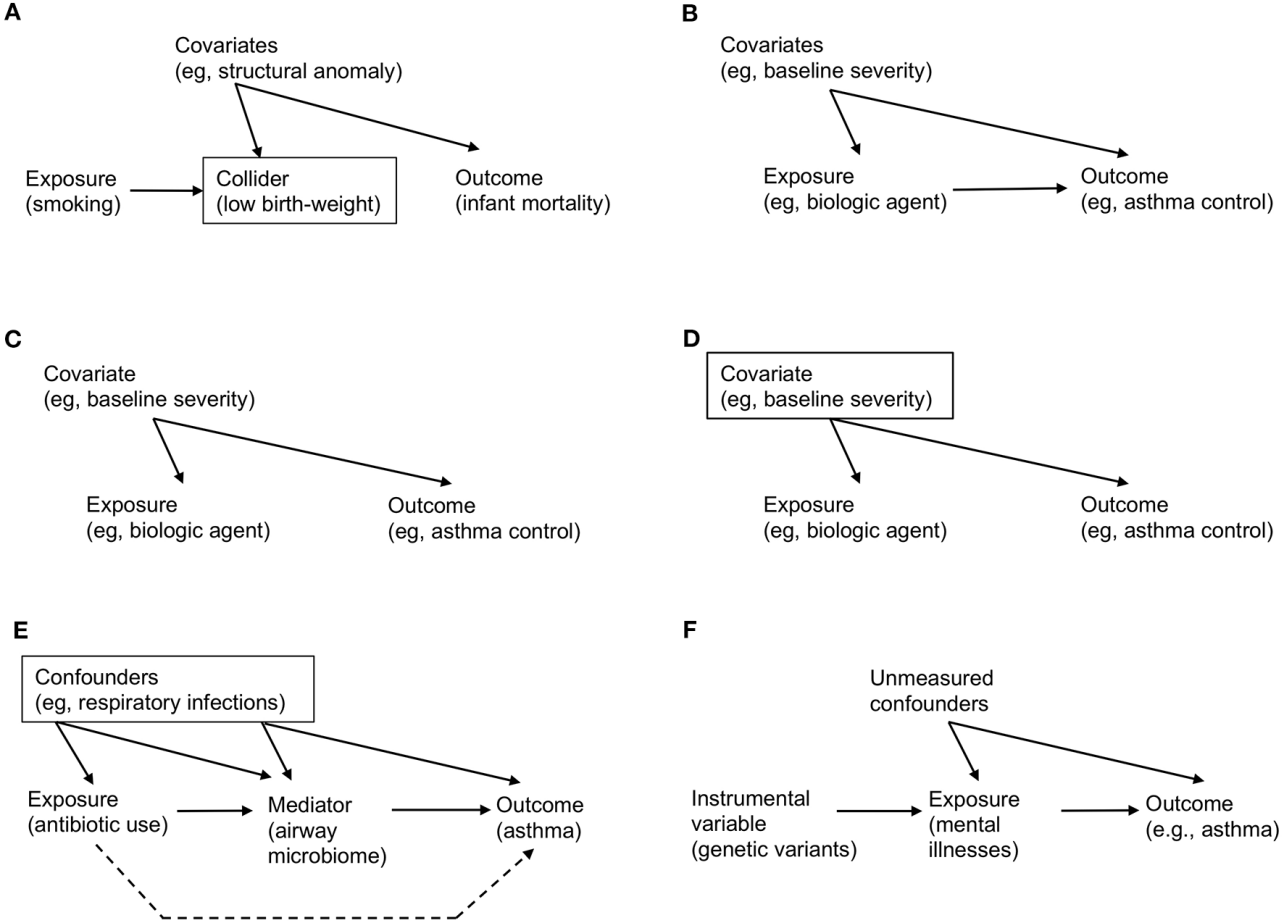
Examples of causal directed acyclic graph that encodes *a priori* domain knowledge and causal structural hypothesis. **(A)** Birth-weight paradox. There is no direct arrow from maternal smoking (exposure) to infant mortality (outcome), representing no causal effect. However, association/prediction-mode machine learning algorithm would automatically adjust for variables that are associated both with smoking and mortality (e.g., low birth-weight). Graphically, a rectangle placed around the low-birth weight variable represents adjustment. However, this adjustment for the collider (a node on which two directed arrows “collide”; Table 1) opens the flow of association from exposure → collider → covariates (e.g., structural anomaly) → outcome, which leads to a spurious (non-causal) association. **(B)** Simple example of causal diagram, consisting of exposure (biologic agent), outcome (asthma control), and covariates (e.g., baseline severity of illness). The presence of edge from a variable to another represents our knowledge on the presence of a direct effect. **(C)** Example of confounding. While there is no causal effect (i.e., no direct arrow from exposure to outcome), there is an association between these variables through the paths involving a common-cause covariate (i.e., a confounder), leading to a non-causal association between the exposure and outcome (i.e., confounding; Table 1). **(D)** Example of de-confounding. This confounding can be addressed by adjusting for the confounder by blocking the back-door path. Graphically, a rectangle placed around the confounder blocks the association flow through the back-door path. **(E)** Example of mediation. The causal relation between the exposure (systemic antibiotic use), mediator (airway microbiome), and outcome (asthma development). The confounders (e.g., acute respiratory infections) between the exposure, mediator, and outcome should be adjusted. The indirect (or mediation) effect is represented by the path which passes through the mediator. The direct effect is represented by the path which does not pass (the broken line; Table 1). **(F)** Example of mendelian randomization. Genetic variants that are strongly associated with the exposure of interest (mental illnesses) function as the instrument variable. Note that there is no association (or path) between the genetic variants and unmeasured confounders (i.e., independent condition) and that the genetic variants affect the outcome only through their effect on the exposure (i.e., exclusion restriction condition; Table 3).

For example, consider the effect of maternal smoking on infant mortality. Data-driven algorithms—which do not encode domain knowledge on the causal structure—will learn from data and fit a curve (very well) by using variables that are strongly associated with maternal smoking and mortality (e.g., infant's birth-weight). However, this automated adjustment for (or stratification by) birth-weight—a potential collider (Figure 1A)—results in a spurious correlation. Specifically, among infants with a low birth-weight, the adjusted risk of mortality is *lower* for those born to smokers [“the birth-weight paradox” (30)]. Alternatively, it is also possible that birth-weight serves as a mediator in the causal path. Adjustment for birth-weight could inappropriately block the causal path, thereby leading to biased inference. Causal effects cannot be quantified by systems that operate exclusively in data-driven association modes, as do most machine learning algorithms today (1). That is, we cannot answer causal questions with the data alone, *no matter* how big the data are and how deep the neural network is.

## Major Causal Inference Tools

Building on the counterfactual framework, epidemiologists, statisticians, and data scientists have developed methods to quantify causal effects from observational data. Table 3 summarizes the major tools, their assumptions (i.e., what to look for), advantages, and disadvantages. These tools enable us to explicitly express causal questions, transparently encode our causal knowledge, and leverage data to consistently estimate the causal effect of interest. Here, we introduce several relevant examples in simplified scenarios.

### Causal Diagram: Codifying Causal Assumptions and De-confounding

Causal diagram is an intuitive graphical tool for qualitatively encoding our domain knowledge and *a priori* assumptions on the causal structure of interest (Tables 1, 3) (15). In other words, it qualitatively models how the cause-effect forces operate and generate data. Stemming from the graph theory developed by the 1700s mathematician Leonhard Euler, its modern tools for causal inference originate from the disciplines of computer science and artificial intelligence.

Consider the following hypothesized causal structure of treatment (a biologic agent), outcome (asthma control), and covariates (the baseline severity of illness) in a causal directed acyclic graph (causal DAG; Figure 1B). It consists of three nodes and three edges. The presence of “edge” from a variable (e.g., biologic agent) to another (e.g., asthma control) means that we know that there exists a direct effect. In contrast, its absence indicates that we know that the biologic agent has no direct effect on asthma control for any individual in the population. In addition to the expressed knowledge, these causal diagrams also encode information on the expected *associations* (more precisely, their absence) between the variables. Unlike causation, an association is a symmetric relationship between two variables. Therefore, an association flows the path between the variables, regardless of the direction of edge. For example, in Figure 1C, even if there is no causal treatment effect (i.e., no direct edge from biologic agent to asthma control), there is an association between these variables through the path involving the severity covariate [i.e., “back-door path” (15)]. The advancement of causal graphs has enabled us (and machines) not only to encode these assumptions and statistical dependencies/independencies, but also to test whether these are compatible with the data.

Confounding—the bias due to common causes of exposure and outcome—has long been considered the major hurdle in causal inference (15). The term “confounding” originates from Latin *confundere* meaning “blending.” The reason why this word was chosen is apparent from the causal diagram. In Figure 1C, the (null) effect of a biologic agent on asthma control is “blended” by the confounder (baseline severity of illness). This is because patients with greater baseline severity may be more likely to receive treatment but have worse control anyway. The apparent spurious correlation is introduced by the open back-door path (i.e., the path through severity). However, this confounding can be “de-confounded” by blocking the back-door path (Figure 1D, in which a rectangle placed around the confounder blocks the association flow through the path) (15). For example, we fit a regression model “adjusting for the confounder” to estimate the causal effect of treatment in every severity group separately [i.e., outcome regression method (Table 3)]. Then, we can take an average of the effects, weighting each severity group according to its probability, to estimate the average causal effect in the population of interest [i.e., standardization for fixed treatments, g-formula for time-varying treatments (15) (Table 3)].

### Causal Mediation: Search for Mechanism

Another major goal of data science is to better understand the connection (or mechanism) between a known cause and effect. Causal mediation analysis aims to tease apart total effects, mediation or indirect effects (which pass through a mediator) and direct effects (which do not) (Tables 1, 3). Counterfactual causal inference needs to be involved to quantify such intermediate mechanisms (14).

For example, there had been uncertainty about the mediating mechanism(s) through which systemic antibiotic exposures in the early life are linked to subsequent asthma development (31, 32). Recently, a team of clinicians, epidemiologists, and data scientists tested a hypothesis—the effect of antibiotic use on asthma is mediated by the changes in airway microbiome [a highly-functional community of microbes (33)] in a population-based cohort (Figure 1E) (34). Statistical estimation of these effects was not trivial given that the number of data dimensions is large (e.g., the complexity of the microbiome) and the causal structure is complex. However, by combining unsupervised machine learning approaches to overcome “the curse of dimensionality” (16) and causal inference methods to carefully account for various confounders, the researchers identified that part of the antibiotic effect on asthma development was mediated by the change in airway microbiome—a modifiable factor. As presented in this example, causal mediation analyses not only provide better understanding on the disease mechanisms but also present opportunities for the development of new therapeutics targeting modifiable mediators (e.g., modulation of microbiome for asthma prevention).

### Mendelian Randomization: Instrument of Nature

Most causal inference methods require a key unverifiable condition—no unmeasured confounding (Table 1). For example, identifying the effect of mental illnesses on asthma development is a difficult question because of many fixed and time-varying confounders (e.g., genetics, socioeconomic status, treatments) (35). To avoid the effect of bias, social scientists have long been using an alternative method—called instrumental variable estimation, which validly yields causal estimates by replacing the condition above with an alternative set of assumptions (Table 3).

In recent years, the increased availability of large-scale genome-wide association study (GWAS) data from biobanks and large consortiums (13, 35, 36) has accelerated the development of an instrumental variable approach—Mendelian randomization (Tables 1, 3). This approach is based on the random assortment of genotypes transferred from parents to offspring at conception. This Mendel's “law of the independent assortment” enables a study relating the genetic variants for modifiable exposures (e.g., mental illnesses) with health outcomes (e.g., asthma) to mitigate the risk of confounding (17). Accordingly, Mendelian randomization is conceptually analogous to an RCT, of which a random assignment of treatment/exposure is equivalent to a randomly-assorted genotype strongly associated with the exposure (Figure 1F). For example, in a study leveraging GWAS datasets of childhood- and adult-onset asthma, the use of Mendelian randomization demonstrated causal effects of depression on asthma (35). Recently, there has been the rise of publicly-available data that relate genetic variants to many *modifiable* exposures, ranging from physical conditions to biomarkers (e.g., proteins, metabolites) (37, 38). This availability of expanded data sources has informed the search for new targeted therapeutics.

### Heterogeneous Treatment Effects: Differentiating Apples From Oranges

**Figure 2:**
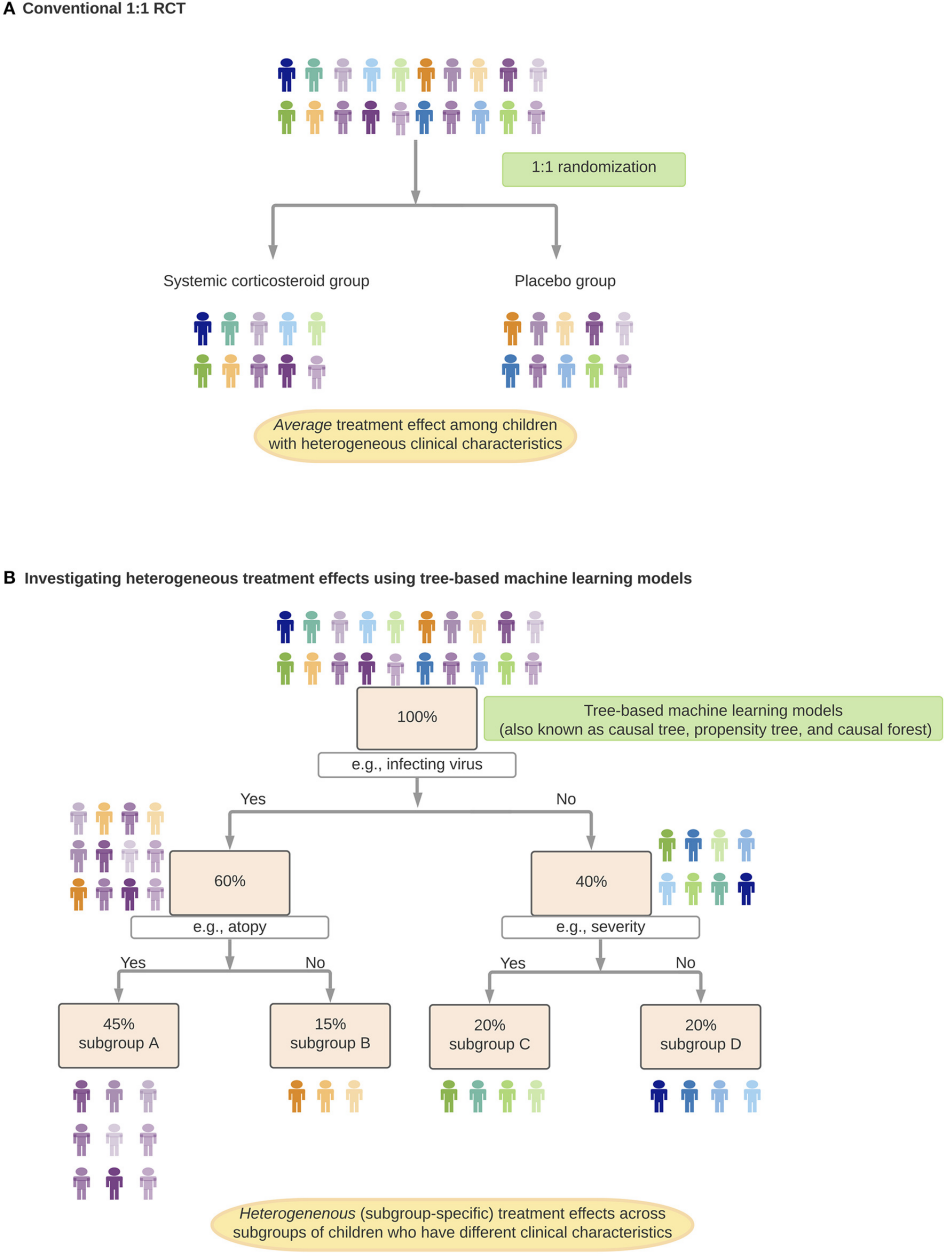
Identification and estimation of heterogenous treatment effects. In this *hypothetical* example, suppose, we investigate treatment effects of systemic corticosteroids on hospitalization rates among preschool children with virus-induced wheezing. **(A)** Randomized control trial (RCT) to investigate the *average* treatment effect of systemic corticosteroids (conventional 1:1 RCT). **(B)** Investigating heterogeneous treatment effects using tree-based machine learning models. In each of the branches (e.g., subgroup A children have specific virus infection and a history of atopy), children have a comparable predicted probability of receiving systemic corticosteroids. Children within each subgroup function as if they came from an RCT with eligibility criteria stratified by clinical characteristics.

RCTs, which have been considered the “gold standard” for causal inference, often attempt to estimate *the* average treatment effect in the target population and generate a uniform recommendation (26). However, it is rare for a treatment effect to be perfectly homogeneous (39). Rather, there often exist effect modifications—either quantitative (i.e., different magnitudes of effects between subgroups) or qualitative (i.e., subgroup[s] having an effect in the opposite direction or no effect) (Table 1) (40). Indeed, growing evidence have shown that various medical disorders are heterogenous [e.g., asthma (13), autism spectrum disorder (41), sepsis (42)] with potentially different underlying mechanisms that lead to differential treatment effects. For example, in preschool children with viral-induced wheezing, most studies have shown no significant *average* effects of systemic corticosteroids on symptom severity or hospitalization rate (43–45). Yet, the question of whether this treatment strategy is beneficial in distinct subgroups of children [e.g., atopic children with rhinovirus-induced wheezing (46)] remains unclear. Recently, machine learning approaches [e.g., random forest (47) (Table 3, Figure 2)] have been applied to health data to (1) identify subgroups with different treatment effects, and (2) estimate individual (heterogeneous) treatment effects for subgroups in various disease conditions (e.g., diabetes) (48, 49). An integration of these algorithms, careful interpretation (e.g., covariate balance between the derived subgroups, false discoveries) and prospective validation will help precision medicine realize preventive and treatment strategies tailored to patients with a unique set of clinical characteristics.

## The Way Forward

### Toward Better Decision-Making and Precision Medicine

A major objective of data science is to assist clinicians and researchers in making better decisions. While its capability is often judged by its success on prediction tasks (11), the proposition that predictive algorithms improve decisions is uncertain. It is important to remember that a data-driven algorithm may excel at predicting, for example, which patients with asthma will be re-hospitalized for asthma exacerbation in the subsequent year, but is agnostic about the reason and possible measures to have prevented it. The algorithm may identify a past emergency department visit for asthma exacerbation as a strong predictor for rehospitalization. However, no clinicians would interpret the emergency department visit as the cause or instruct patients not to present to the emergency department. Identifying patients with a worse prognosis (through prediction) is a different question from identifying the optimal prevention and treatment strategies for a specific group of patients—the defining question of precision medicine (through causal inference). In other words, data-driven prediction algorithms can only point out decisions to be made, whereas causal inference can assist in decision making.

Note that these contrasts between association/prediction and causal inference tasks would become less sharp when the domain knowledge and counterfactual reasoning are codified in algorithms. Within a simple system with known deterministic rules and complete information [e.g., chess and Go games (22)], such algorithm is possible to predict outcomes under any hypothetical intervention (or any hypothetical move). By contrast, clinicians and researchers in the medical fields regularly deal with complex systems governed by non-deterministic rules with uncertainties about available data. Suppose we are interested in the causal effect of a new drug on infants with severe bronchiolitis. We only have incomplete knowledge on the causal structure through which the respiratory viruses, host genetic and immune factors, and environments jointly regulate and/or mediate the effect in this heterogeneous disease condition (50). Accordingly, most clinical researchers and epidemiologists had tended to answer carefully-developed but relatively-narrow causal questions (e.g., *the* average treatment effect of bronchodilators in infants with bronchiolitis) rather than to elucidate the global structure of system which could enable clinicians to make broadly optimal decisions (e.g., heterogeneous treatment effects between different bronchiolitis subgroups with distinct mechanisms).

In the past decade, the integration of “big data” with data science approaches (i.e., machine learning and causal inference equipped with domain knowledge) has begun to challenge conventional views. An example is the recent development of targeted treatment for asthma. There has been a growing consensus that asthma consists of different subtypes (13). Collective evidence from data science, experiments, and RCTs have already enabled clinicians to identify patients with a specific subtype of asthma (e.g., allergic asthma, eosinophilic asthma) by effective biomarkers (e.g., IgE, fractional exhaled nitric oxide, eosinophil quantification) and to provide targeted treatments (e.g., anti-IgE, anti-IL-5 therapies) (51, 52).

Another example is infant bronchiolitis, which is not only the leading cause of infant hospitalization in the U.S. (53) but also one of the strongest risk factors for asthma development (54). While bronchiolitis has been considered a single disease entity with similar clinical characteristics and mechanisms (55), emerging evidence indicates substantial heterogeneity (56–63). Indeed, recent studies applying data science approaches to large bronchiolitis cohorts have identified (62) and validated (63) the presence of different subtypes of bronchiolitis that have a higher risk of developing recurrent wheezing (e.g., atopic infants with rhinovirus infection who present with wheezing, compared to “classic” RSV bronchiolitis). Further, a recent study has also identified biologically-distinct subtypes of bronchiolitis that have higher risks of developing asthma (e.g., infants with type 2 airway inflammation with a dominance by specific virus and bacteria) (58). These efforts driven jointly by data scientists, clinical and laboratory researchers, and clinicians have potential to offer new avenues for developing prevention (e.g., early identification of high-risk children before disease inception) and treatment (subtype-specific treatment at a critical period of organ development) strategies in various disease conditions in children.

### Future Challenges

For the successful development and implementation of data science approaches in clinical practice, several challenges and limitations need to be addressed. First, there are methodological challenges—e.g., how to fulfill standard causal inference assumptions (e.g., consistency when there are non-homogeneous exposures), how to model multiple molecular mediators at multiple levels, and how to handle time-varying feedbacks in a complex system. These are active areas of research. Second, evidence derived from these data science by itself is not confirmatory. We note that its promise lies in their symbiosis with, not replacement of, conventional experimental studies and RCTs. The derivation of novel and well-calibrated hypotheses based on robust data science still require stringent validations and experiments. Each approach can benefit from the other, which will, in turn, advance medical sciences and clinical practice. Lastly, milestones needed for data science-assisted medicine to become a reality go beyond methodological advents. The healthcare structure ought to adapt to operate with inter-disciplinary teams (e.g., clinicians, data scientists, epidemiologists, informatics specialists). Additionally, with the growing gap between the amount of data and clinical expertise, the realization of precision medicine warrants continued education for clinicians who interpret data and translate findings into clinical practice. For clinicians who wish to learn more, Table 4 summarizes educational resources.

**Table 4:** Twelve major resources for clinicians who wish to learn about data science.

**Topic**	**Type**	**Platform/Resource**	**Content summary**
Data science (in general)	MOOC	Kahn academy	An online course that covers a wide range of topics about statistical analyses
	MOOC	Coursera: data science specialization	An online course that provides a broad overview of data science
	MOOC	edX: introduction to probability (HarvardX STAT110x)	An online course that introduces the basics of probability theories, which are fundamental for data science, statistics, and causal inference
	MOOC	Stanford: statistical learning	An online learning course that offers an introduction to various statistical learning (including machine learning) approaches
	Textbook	*An Introduction to Statistical Learning*	A well-written introductory textbook that is used in the statistical learning course (see above)
	Paper	*BMJ*: research methods & reporting	*BMJ* series introduces important topics of epidemiology and biostatistics to help clinicians interpret the medical literature
	Paper	*JAMA*: guide to statistics and medicine	*JAMA* series introduces important statistical techniques to help clinicians interpret the medical literature
Machine learning	MOOC	Coursera: machine learning	One of the most popular machine learning courses (as of January 2021, 3.9 million students have been enrolled). This introductory course provides an overview of various machine learning algorithms
	MOOC	Coursera: Deep learning specialization	A more detailed online course that covers the basics and applications of various deep learning algorithms
Causal inference	MOOC	edX: Causal diagrams (HarvardX PH559x)	An online course that introduces an overview of causal diagrams in clinical research
	MOOC	Coursera: A crash course in causality	An online course offered that provides an introductory overview of causal inference theories and approaches
	Textbook	*Causal Inference in Statistics: A Primer* (64)	Introductory-level textbook that covers important topics in causal inference (e.g., causal diagram)
	Textbook	*Causal Inference: What if* (15)	Comprehensive intermediate-level textbook that provides the concepts of and methods for causal inference in clinical research
Programming	MOOC	Coursera: foundations using R specialization	An online course that provides a broad overview of R programing
	Others	DataCamp	A collection of introductory video lectures and hand-on coding practices in several programing languages (e.g., R, python)

*MOOC, massive open online course; BMJ, British Medical Journal; JAMA, Journal of the American Medical Association.*

*All of the listed MOOCs are publicly-available without fee*.

### Summary

In this review, we summarize the goals, tasks, and tools of data science. Data science is a component of scientific disciplines, including epidemiology and medicine. Thus, the tasks of data science are the tasks of those disciplines—i.e., association/prediction, intervention, and counterfactual causal inference.

**Figure 3:**
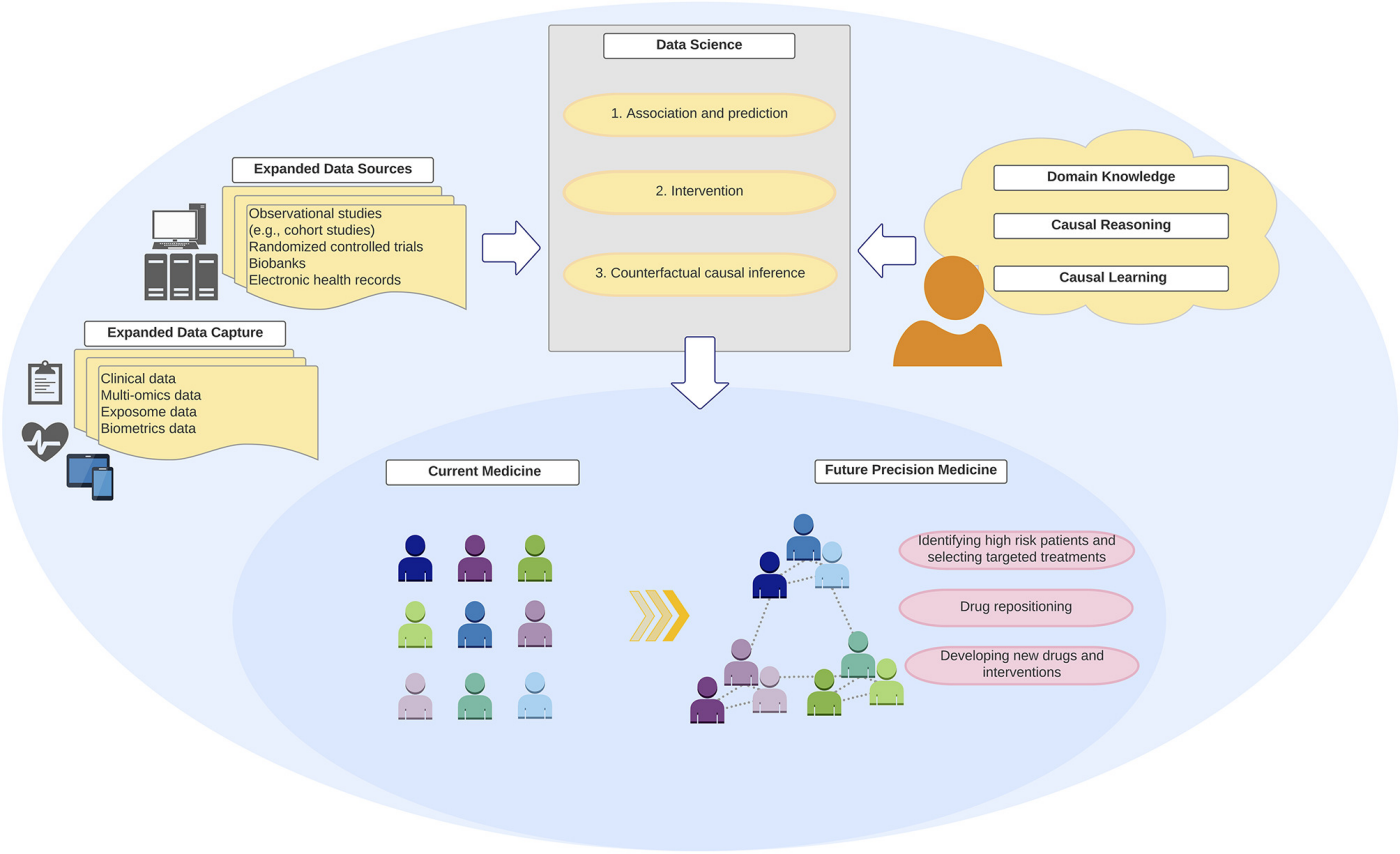
Integration of “big data,” data science, and domain knowledge toward precision medicine. Development of precision medicine requires an integration of “big data” from expanded data sources and capture with robust data science methodologies and analytics that encode domain causal knowledge and counterfactual causal reasoning.

In this “big data” era, clinical practice and research have called for clinicians and researchers to handle a growing amount of data—e.g., clinical, biometric, and biomarker data. While machine learning algorithms become ubiquitous tools to handle quantitatively “big data,” their integration with domain knowledge and causal reasoning is critical to understand how complex systems behave (Figure 3). This integration in data science is key to qualitatively transform medicine. Patients—whose lives shape data, knowledge, and algorithms—will benefit the most as this new scientific discipline advances precision medicine.

## Author Contributions

YR and KH conceptualized and developed the primer, drafted the initial manuscript, and reviewed and revised the manuscript. LL and CC conceptualized and supervised the development of the primer, and reviewed and revised the manuscript. All authors approved the final manuscript as submitted and agree to be accountable for all aspects of the work.

## Conflict of Interest

The authors declare that the research was conducted in the absence of any commercial or financial relationships that could be construed as a potential conflict of interest.

## Abbreviations

ED: emergency department
GWAS: genome-wide association study
RCT: randomized controlled trials.
